# Molecular determinants of lung function decline: a multi-level analysis of gene expression

**DOI:** 10.1186/s12931-025-03450-z

**Published:** 2025-12-17

**Authors:** Zaid W. Elhusseini, Omar Rafique, Min Hyung Ryu, Peter Castaldi, Don D. Sin, Ingo Ruczinski, Craig P. Hersh

**Affiliations:** 1 Channing Division of Network Medicine, Brigham and Women’s Hospital, Harvard Medical School, 181 Longwood Avenue, Boston, MA USA; 2 Division of Respiratory Medicine, Faculty of Medicine, University of British Columbia, Vancouver, Canada; 3 Department of Biostatistics, Johns Hopkins Bloomberg School of Public Health, Baltimore, USA; 4 Division of Pulmonary and Critical Care Medicine, Brigham and Women’s Hospital, Boston, MA USA

**Keywords:** COPD, Pulmonary function tests, RNA-sequencing, Longitudinal analysis, Biomarkers

## Abstract

**Background:**

Chronic obstructive pulmonary disease (COPD) is characterized by progressive lung function decline, commonly measured by forced expiratory volume in one second (FEV_1_). Uncovering the genetic basis of FEV_1_ decline is essential for understanding COPD pathophysiology and for developing therapies. We hypothesized that gene expression patterns in inflammatory pathways are associated with FEV_1_ decline.

**Methods:**

We analyzed whole blood RNA-sequencing data from the 5 (*n* = 4,147) and 10 year visits (*n* = 435) in the COPDGene Study. Gene expression was assessed in three analyses: cross-sectional associations with FEV_1_ at two separate time points, association between year 5 gene expression and FEV_1_ changes from year 5–10, and longitudinal changes in both gene expression and FEV_1_. A gene signature derived from the 5-year visit was linked to FEV_1_ decline across three intervals (baseline to 5 years, 5 to 10 years, and baseline to 10 years) and tested for validation in the ECLIPSE study.

**Results:**

Distinct gene sets emerged in the three analyses (Cross-sectional: 961 genes; FEV_1_ Change: 179; Longitudinal: 532). Only two genes (*NOV* and *AC009404.2*) overlapped across all analyses, while unique genes (e.g., *MMP9*, *IL1RL1*, and *CHI3L1*) were context-specific. Pathway analysis of genes from the longitudinal analysis highlighted oxidative stress and immune processes. A 20-gene signature was derived, including 17 genes positively and three negatively associated with FEV_1_. These signatures were significantly associated with FEV_1_-related traits in COPDGene and ECLIPSE.

**Conclusions:**

These findings reveal molecular markers of FEV_1_ decline, offering insights into COPD pathophysiology and potential therapeutic targets.

**Supplementary Information:**

The online version contains supplementary material available at 10.1186/s12931-025-03450-z.

## Background

Chronic Obstructive Pulmonary Disease (COPD) is a heterogeneous disease characterized by persistent respiratory symptoms and airflow limitation. Major causes include environmental factors such as exposure to cigarette smoke and pollutants, together with molecular factors such as alpha-1 antitrypsin deficiency [1]. The forced expiratory volume in 1 s (FEV_1_) on spirometry is a widely recognized and critical marker used for diagnosing and monitoring the severity of COPD [2]. Studies show that FEV_1_ decline correlates with exacerbations and mortality risk [3]. Longitudinal analyses have shown that an accelerated decline in FEV_1_ is a key factor in COPD development, with research indicating that up to 50% of cases arise from this progressive decline [4]. Thus, understanding factors that contribute to FEV_1_ decline is vital for the effective management of COPD [5].

While lifestyle and environmental exposures, such as continued smoking and repeated exacerbations, are significant contributors, the mechanisms driving COPD progression are still poorly understood. Growing evidence suggests that molecular factors play a crucial role in disease progression, particularly through their effect on lung function decline [6]. Utilizing large longitudinal studies like the Genetic Epidemiology of COPD Study (COPDGene) with clinical and RNA-sequencing data, provides an opportunity to explore these mechanisms more deeply.

We hypothesized that gene expression patterns are associated with subsequent FEV_1_ decline and may serve as early biomarkers of COPD progression. To test this, we used models assessing the association between gene expression and future FEV_1_ decline, as well as within-subject changes over time. Combining insights from these approaches provides a more comprehensive understanding of COPD progression, enabling the identification of molecular underpinnings and potential therapeutic targets [6].

## Methods

### COPDGene study

COPDGene is a longitudinal prospective study that recruited 10,192 smokers with at least a 10-pack-year smoking history, aged between 45 and 80 years, including subjects with and without COPD at enrollment [7]. Clinical assessments and questionnaires were collected at enrollment, with follow-up visits occurring 5 (Phase 2) and 10 years (Phase 3) after enrollment. Blood samples were collected during the second and third visits for RNA sequencing. Additional details are provided in the online supplement.

### Gene expression

COPDGene RNA-sequencing methods were reported previously [8, 9]. RNA-seq data from whole blood samples of 4,147 participants in Phase 2 and 511 participants in Phase 3 were retained for analysis after quality control. Phase 3 subjects were randomly selected for RNA-seq; this analysis represents the first data freeze.

### Data processing and linear regression analysis

**Fig. 1:**
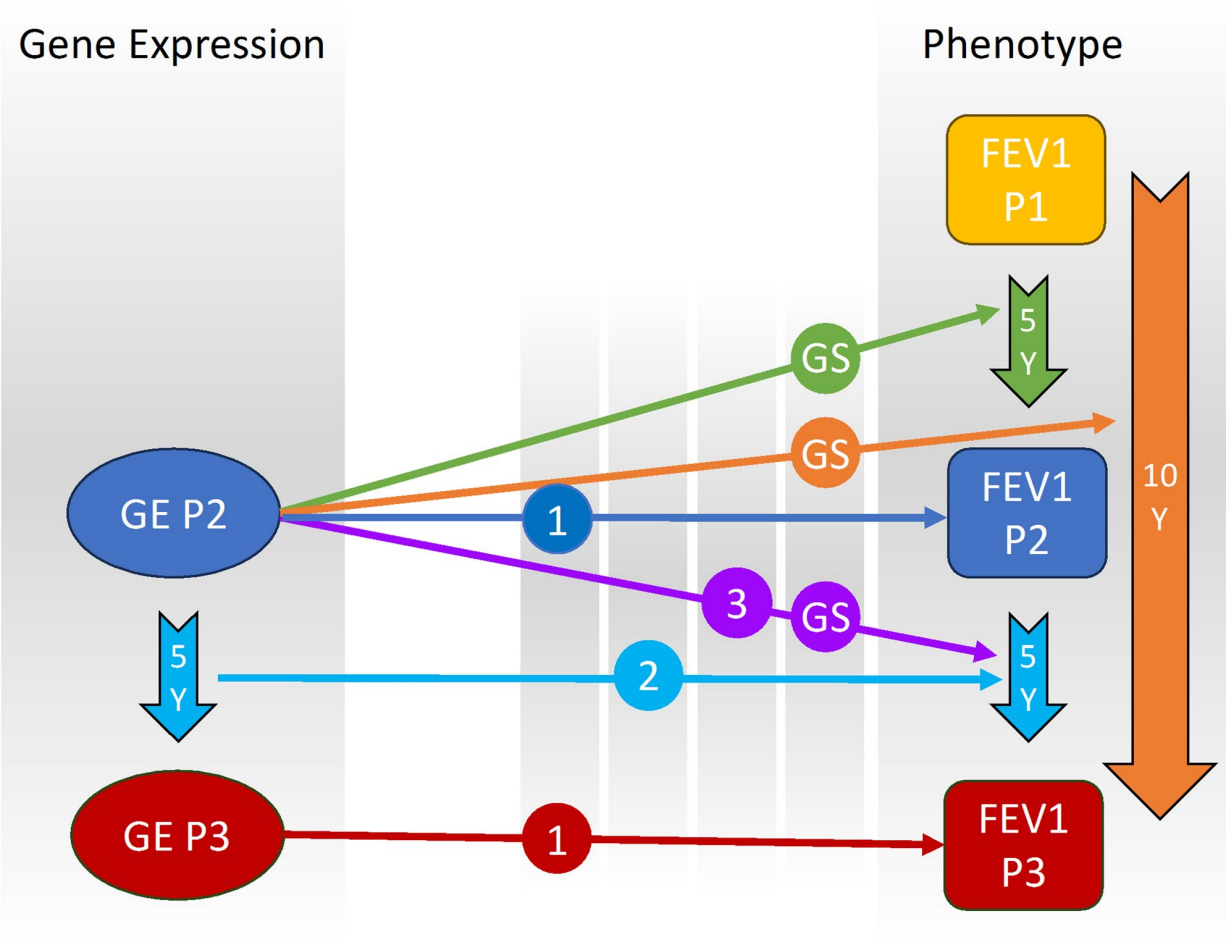
Multi-phase analysis of FEV_1_ in COPDGene. (1) Cross-sectional analysis: Association between gene expression (GE) in Phase 2 and Phase 3 with FEV_1_ in Phase 2 and Phase 3, respectively. (2) Longitudinal gene expression change analysis: Association between the change in gene expression from Phase 2 to Phase 3 and the change in FEV_1_ from Phase 2 to Phase 3. (3) FEV_1_ change analysis: Association between gene expression in Phase 2 and the change in FEV_1_ from Phase 2 to Phase 3. (GS) Gene signature generation: Combining the three analyses to create a gene signature associated with FEV_1_ change by examining gene expression in Phase 2 and FEV_1_ change across all three intervals. FEV_1_, forced expiratory volume in one second; P, Phase; Y, Years

We performed linear regression analyses to evaluate the relationship between gene expression and lung function decline, focusing primarily on FEV_1_ change and longitudinal models (Fig. 1, Supplemental Table 1). The FEV_1_ change analysis used gene expression data from Phase 2 along with the change in FEV_1_ for three intervals: Phase1-Phase2, Phase2-Phase3, and Phase1-Phase3. For the longitudinal analysis, we assessed the changes of both Phase 2 and Phase 3 FEV_1_ and gene expression data.

For the longitudinal analysis, we combined gene expression data from both phases, ensuring that only samples present in both phases were included. Batch effects were adjusted using ComBat-seq [10]. Samples belonging to unique batches were excluded since the batch correction method does not accept single-batch samples. We retained genes where at least 80% of samples had counts per million (CPM) > 1. We then normalized the data using the TMM method and applied the voom transformation. In the longitudinal analysis, we calculated the difference in gene expression between the two-time points. Finally, we performed a linear model in Limma [11] which enables precise variance estimation and covariate adjustment in large sample sizes, adjusting for age, race, sex, smoking status (current or former), and white blood cell count and differential percentages.

### Pathway enrichment analysis

We performed pathway enrichment analysis using the 532 genes identified with *p* < 0.05 in the longitudinal analysis. These genes were submitted to g: Profiler (https://biit.cs.ut.ee/gprofiler). The data sources used for this analysis included Gene Ontology (Biological Process), KEGG, Reactome, and WikiPathways. To identify significant pathways, we applied the g: SCS threshold, which adjusts for multiple testing to control the family-wise error rate. The resulting pathways were ranked based on their adjusted p-values.

### FEV_1_ change gene signature

To reduce multiple testing burden for association with other COPD phenotypes, generated a gene signature associated with FEV_1_ change by performing linear regression analysis. We included all subjects with FEV_1_ readings across three intervals (Phase 1 to Phase 2, Phase 2 to Phase 3, and Phase 1 to Phase 3) and identified genes with a nominal p-value < 0.05 for each interval. The gene signature was determined by identifying the overlapping genes across all three intervals. Genes were separated based on the direction of their effect into a positive and negative gene signature. We then performed Gene Set Variation Analysis (GSVA) [12] to assign each subject a score based on these gene sets. We then assessed the association between the gene signature and COPD-related traits, using linear or logistic regression analysis, adjusting for age, race, sex, and smoking status, as well as white blood cell counts and differential percentages.

### Validation in ECLIPSE study

The ECLIPSE Study was a longitudinal prospective study involving 2,747 participants with at least a 10-pack-year smoking history [13], aged between 40 and 75 years. Study visits occurred at enrollment, three months, and every six months until 3 years. Data collected included spirometry, questionnaires, and other clinical assessments. Gene expression microarray (Affymetrix Human Gene 1.1 ST array) data are available for 627 peripheral blood samples from both COPD and control subjects [13].

## Results

**Fig. 2:**
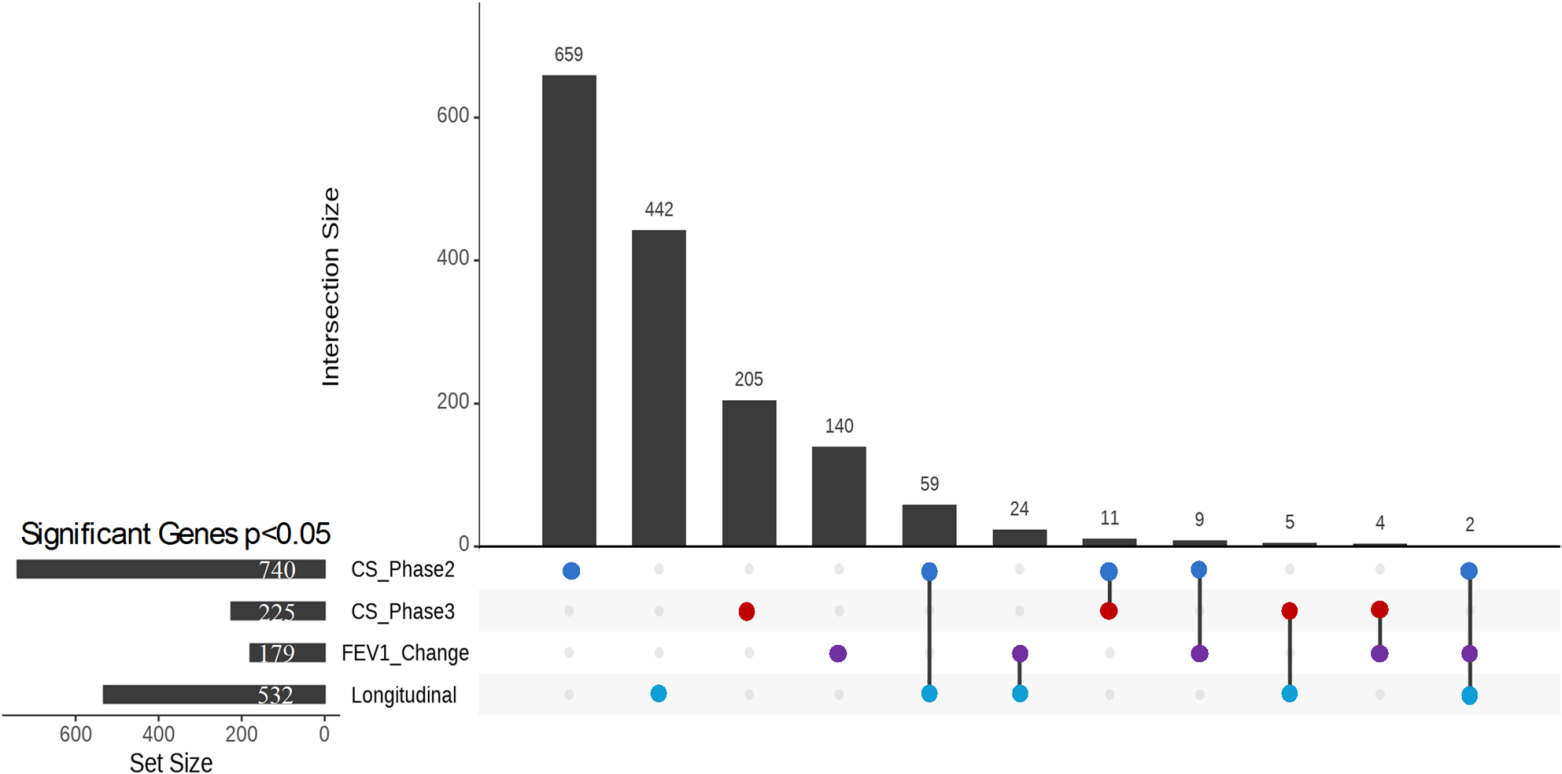
Upset plot of Overlapping Genes Across Cross-Sectional and Longitudinal Analyses. See Fig. 1 for descriptions of analyses. CS, Cross-Sectional; FEV_1_, forced expiratory volume in one second

We selected 435 subjects who had both RNA-sequencing and phenotype data in Phase 2 and Phase 3 (Table 1). While the primary focus was on longitudinal and FEV_1_ change analyses, we also conducted cross-sectional analyses at Phases 2 and 3 for context; full results are reported in the Supplementary Materials (Supplemental Tables 2–3). For the FEV_1_ change analysis, where we tested the association between gene expression in Phase 2 and the change in FEV_1_ from Phase 2 to Phase 3, we identified 179 genes at *p* < 0.05 (Supplemental Table 4). Finally, in the longitudinal analysis, where we assessed the association between the change in gene expression and the change in FEV_1_, we found 532 genes at *p* < 0.05 (Supplemental Table 5). Although several genes reached nominal significance (*p* < 0.05) in the FEV_1_ change and longitudinal analyses, none passed FDR correction, likely due to smaller sample sizes and the complexity of modeling intra-individual lung function change. To explore the overlap between these analyses, we examined the intersections of genes identified in FEV_1_ change, longitudinal, and supplementary cross-sectional analyses. Interestingly, only two genes (*NOV* and *AC009404.2*) were shared across all three analyses, suggesting a limited but potentially critical set of common molecular determinants (Fig. 2). Additionally, each analysis revealed unique sets of genes previously related to COPD (Supplemental Table 6), cross-sectional (*TNF*,* TLR4*,* IL6R*), FEV_1_ change (*CHI3L1*,* VEGFA*,* FOXO3*), and longitudinal (*MMP9*,* IL1RL1*,* ALOX5AP*), highlighting context-specific markers of lung function decline. These results demonstrate the importance of combining cross-sectional and longitudinal approaches to capture dynamic changes in gene expression associated with lung function decline.

**Table 1:** Characteristics of the study population

*N* = 435	Units	Phase 2				Phase 3		
		Mean	SD	Range		Mean	SD	Range
Age	Years	64.52	8.17	48.1–84.6		69.19	8.28	53-89.4
FEV_1_ post-bronchodilator	Liters	2.31	0.76	0.4–4.44		2.12	0.75	0.32–4.36
FEV_1_% predicted	%	84.26	22.59	14.7-137.6		82.34	24.24	12.7–144.2
Neutrophils	%	58.26	9.56	25–91		60.02	9.64	19.3–93.9
Lymphocytes	%	30.5	9.03	7.0–59		28.25	9.27	2.6–78.9
Eosinophils	%	2.57	1.98	0–25		2.5	1.65	0-8.8
Monocytes	%	8.04	2.53	1–22.0		8.43	2.39	0–22
WBC	K/uL	6.84	1.98	2.8–18.9		6.83	1.97	2.3–22.6
ΔFEV_1_(Phase2-3)	ml/year	-40.98	56.46	-406–179				
Pack Years of smoking		44	23.69	10–145		44.94	23.95	10–145
Phase2						Phase3		
* N* = 435	Units					*N* = 435	Units	
Sex (Male)	%	49				Sex (Male)	%	49
Former Smokers	%	67				Former Smokers	%	70
Race (non-Hispanic White)	%	74				Race (White)	%	74
Race (African American)	%	26				Race (African American)	%	26
Smoking status in phase 2–3	FF	FC	CF	CC				
	278	12	25	120				

*FEV*_1_ Post-BD, *FEV*_1_ Post bronchodilator, *FF* Former smoker in Phase2 and Phase3, *CC* Current smoker un Phase2 and Phase3, *CF* Current smoker in Phase2 and Former smoker in Phase3, *FC* Former smoker in Phase2 and Current smoker in Phase3

We then conducted a pathway enrichment analysis on the 532 genes identified at *p* < 0.05 in the longitudinal gene-expression analysis (Supplemental Table 7). This analysis identified significantly associated pathways related to hemoglobin production and erythrocyte function, including heme biosynthesis, hemoglobin metabolic process, and erythrocyte differentiation. Additionally, pathways such as myeloid cell differentiation and immune system processes were enriched.

**Fig. 3:**
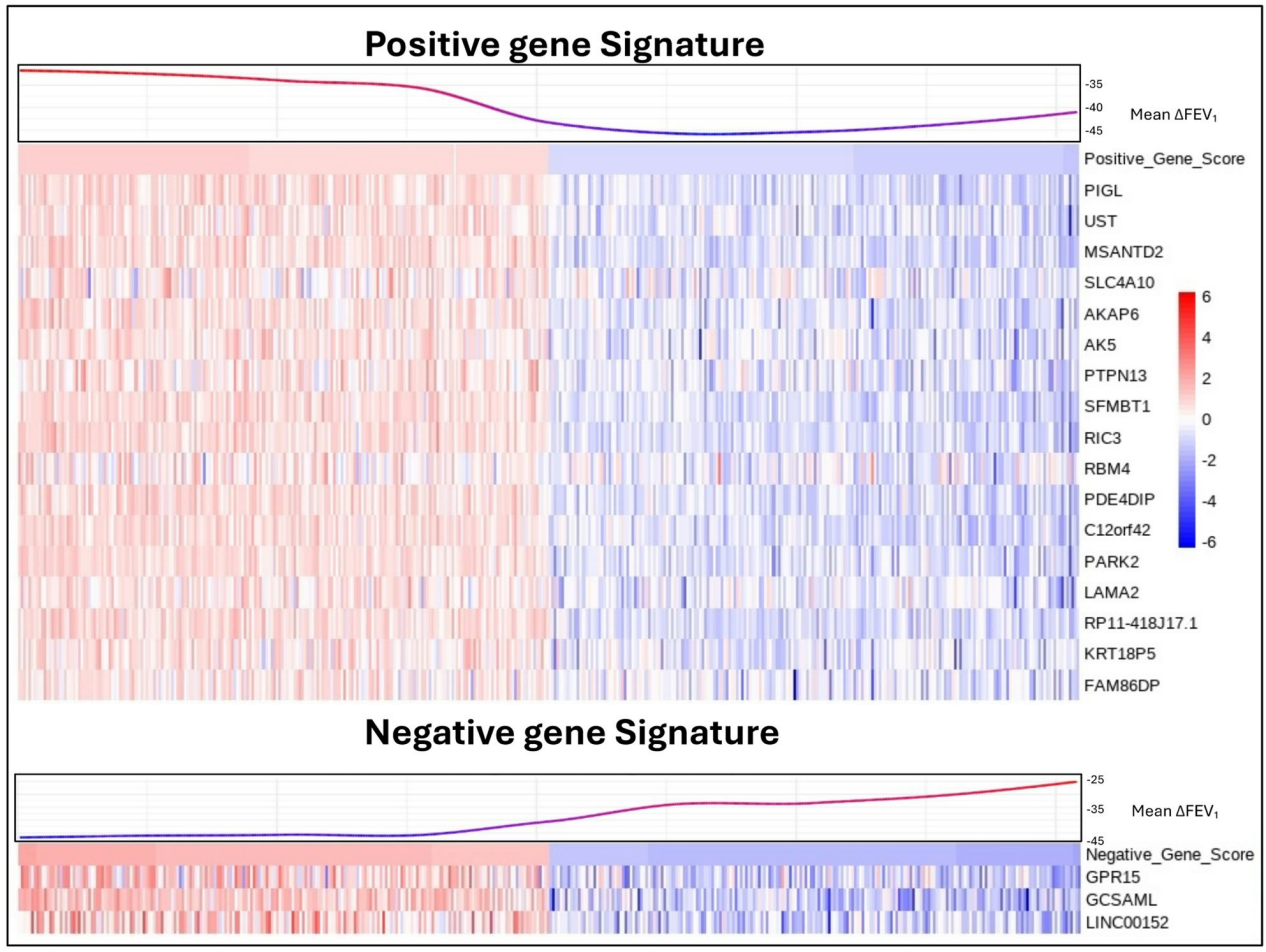
Heatmap of FEV_1_ change Gene Signatures. Subjects are sorted based on their GSVA scores for the gene signatures, with the top 10% of subjects from each end of the GSVA score distribution selected for the figure. Non-linear LOESS regression was applied to the mean FEV_1_ change values across three intervals—Phase1-Phase2, Phase2-Phase3, and Phase1-Phase3—for each subject. The heatmap represents the normalized gene expression data for each subject, with values scaled by row to highlight relative gene expression differences across the cohort. ΔFEV_1_, forced expiratory volume in one second change

We conducted linear regression analyses to assess the relationship between gene expression in Phase 2 and FEV_1_ change across three intervals. In the Phase 1 to Phase 2 interval, 3,805 subjects with both phenotype and RNA-seq data were analyzed, resulting in 1,545 genes with *p* < 0.05 and 26 genes meeting FDR < 0.05 (Supplemental Table 8). For the Phase 2 to Phase 3 interval, 2,045 subjects were included, resulting in 608 genes with *p* < 0.05, though none passed FDR < 0.05 (Supplemental Table 9). Lastly, in the Phase 1 to Phase 3 interval, 2,037 subjects were analyzed, resulting in 521 genes with *p* < 0.05, with 1 gene (*GPR15*) passing FDR < 0.05 (Supplemental Table 10). To generate a gene signature associated with FEV_1_ change, we identified all overlapping genes with a nominal p-value < 0.05 across the three intervals, taking into account their fold change direction. This approach resulted in 17 genes positively associated with FEV_1_ (Positive Gene Signature) and 3 genes negatively associated with FEV_1_ (Negative Gene Signature). We then assigned each patient a score based on the expression of these two gene signatures using GSVA (Fig. 3).

Instead of testing individual genes, we tested the association of the two composite gene signatures with phenotypes related to lung function, finding significant associations with FEV_1_ in Phase 2 (the same year as gene expression collection) and Phase 3 (five years later), rapid FEV_1_ decline (defined as more than 40 ml/yr) [14], and COPD case-control status (Table 2). The positive and negative gene signatures were associated with other COPD-related traits, including exacerbation frequency in the previous year, severe exacerbations requiring hospitalization or emergency department visits, and chest CT measurements of percent emphysema (LAA 950) and airway wall thickness (Pi10) [15]. Both signatures showed a direction of association aligning with the known association between FEV_1_ and the respective trait. To evaluate the clinical utility of the gene signatures, we performed receiver operating characteristic (ROC) analysis to predict rapid FEV_1_ decline. While both GS_Pos and GS_Neg marginally increased the area under the ROC curve compared to covariates alone, the improvement was not statistically significant (Supplementary Figure S1).

**Table 2:** FEV_1_ decline gene signature association with COPD-related traits in COPDGene

Traits	Positive Gene Signature			Negative Gene Signature				Regression
	Beta	95% CI	*P*.value	Beta	95% CI	*P*.value		
**FEV**_**1**_ **at Phase 2 (ml)**	310.82	[241.86, 379.78]	1.47E-18	-170.17	[-216.15, -124.19]	4.79E-13	Linear	
**FEV**_**1**_ **at Phase 3 (ml)**	310.35	[219.98, 400.72]	2.13E-11	-161.43	[-222.01, -100.85]	1.91E-07	Linear	
**FEV**_**1**_ **Rapid Decline Phase1_Phase2 (< 40 ml/yr)**	-0.27	[-0.48, -0.06]	1.17E-02	0.23	[0.09, 0.37]	1.20E-03	Binary	
**FEV**_**1**_ **Rapid Decline Phase2- Phase 3 (< 40 ml/yr)**	-0.38	[-0.66, -0.09]	8.92E-03	0.29	[0.10, 0.48]	2.64E-03	Binary	
**FEV**_**1**_ **Rapid Decline Phase1- Phase3 (< 40 ml/yr)**	-0.69	[-0.98, -0.40]	3.02E-06	0.36	[0.17, 0.55]	2.68E-04	Binary	
**COPD case-control (Gold Stage 0 VS 2–4)**	-1.06	[-1.32, -0.81]	3.41E-16	0.75	[0.58, 0.92]	1.03E-17	Binary	
**COPD related Traits**								
**Airway Wall Thickness (Pi10)**	-0.15	[-0.21, -0.09]	1.50E-06	0.13	[0.09, 0.17]	1.16E-10	Linear	
**Severe Exacerbations**	-1.18	[-1.56, -0.81]	9.94E-10	0.33	[0.09, 0.58]	8.17E-03	Binary	
**Exacerbation Frequency**	-0.26	[-0.34, -0.18]	2.76E-10	0.08	[0.03, 0.13]	4.01E-03	Linear	
**% emphysema (-950HU)**	-2.78	[-3.68, -1.87]	1.84E-09	1.81	[1.21, 2.41]	4.01E-09	Linear	
**Change P1 to P2: Emphysema**	-0.77	[-1.18, -0.36]	2.12E-04	0.47	[0.20, 0.75]	6.34E-04	Linear	

*GOLD* Global initiative for chronic Obstructive lung Disease, *FEV*_1_, Forced expiratory volume in 1 s; Pi10, The square root of the wall area of a hypothetical airway with a 10-mm internal perimeter

Finally, we aimed to validate our findings in the ECLIPSE study by testing the association between the two gene signatures and FEV_1_ measurements at baseline and follow-up visits, FEV_1_ decline, exacerbation frequency, percent emphysema, and Pi10. Both signatures showed significant associations with FEV_1_ at baseline and follow-up, as well as with the percent emphysema and exacerbation frequency (Table 3). However, we were unable to find a significant association between the gene signatures and the change in FEV_1_ (ml/yr).

**Table 3:** Validation of FEV_1_ decline gene signature association with COPD-related traits in the ECLIPSE study

Traits	Positive Gene Signature			Negative Gene Signature			Regression
	Beta	95% CI	*P*.value	Beta	95% CI	*P*.value	
**FEV**_**1**_ **at baseline**	360.24	[187.29, 533.20]	4.89E-05	-352.34	[-476.43, -228.24]	3.71E-08	Linear
**FEV**_**1**_ **at 1 year**	333.50	[161.11, 505.89]	1.60E-04	-300.79	[-426.23, -175.36]	3.09E-06	Linear
**FEV**_**1**_ **at 2 years**	329.83	[156.85, 502.80]	1.98E-04	-312.89	[-438.13, -187.64]	1.20E-06	Linear
**FEV**_**1**_ **at 3 years**	294.18	[112.63, 475.73]	1.54E-03	-327.72	[-459.47, -195.98]	1.35E-06	Linear
**FEV**_**1**_ **Change ml/yr**	4.68	[-15.69, 25.04]	6.52E-01	-2.50	[-17.51, 12.50]	7.43E-01	Linear
**% emphysema (-950HU)**	-4.49	[-7.77, -1.20]	7.49E-03	4.28	[1.94, 6.63]	3.59E-04	Linear
**Exacerbation Frequency**	-0.73	[-1.13, -0.34]	3.10E-04	0.03	[-0.26, 0.32]	8.54E-01	Linear
**Airway Wall Thickness (Pi10)**	-0.02	[-0.05, 0.02]	2.89E-01	0.02	[-0.03, 0.06]	4.39E-01	Linear

*FEV*_1_, Forced expiratory volume in 1 s; Pi10, The square root of the wall area of a hypothetical airway with a 10-mm internal perimeter

## Discussion

We performed RNA-sequencing on whole blood samples collected at Phase 2 (5-year) and Phase 3 (10-year) visits in the COPDGene Study to identify gene expression signatures associated with FEV_1_ decline. Our primary analyses included FEV_1_ change and longitudinal models; cross-sectional findings are presented in the Supplementary Materials to provide additional context. Each analysis revealed a unique set of genes, and there was a limited overlap between the results, highlighting the complexity of gene expression changes over time in COPD. In addition, we generated a gene signature relevant to lung function decline that is significantly associated with key clinical traits related to COPD. Finally, we were able to replicate most of the gene signature associations in the ECLIPSE study, demonstrating the robustness of the composite signatures.

We compared the outcomes of the FEV_1_ change analysis with the cross-sectional analysis. We found that 15 genes (*p* < 0.05) overlapped between the FEV_1_ change analyses and the cross-sectional analysis. Additionally, 137 genes were uniquely associated with FEV_1_ change and not identified in the other analyses. These findings suggest that while some genes are consistently associated with FEV_1_ in both types of analysis, other genes may be more relevant for changes in lung function over time.

Finally, when we compared these previous analyses with the longitudinal linear regression analysis, which examined the association between changes in gene expression from Phase 2 to Phase 3 and the change in FEV_1_ over the same period, we observed several overlaps. Two genes were common to both the cross-sectional and FEV_1_ change analyses, 64 genes overlapped only with the cross-sectional analysis, 24 genes were shared exclusively with the FEV_1_ Change analysis, and 442 genes were unique to the longitudinal analysis. These results suggest that the longitudinal analysis uncovers additional gene associations that may be missed by cross-sectional or FEV_1_ change approaches, emphasizing the value of longitudinal methods in identifying genes related to changes in lung function.

Among the genes identified in the longitudinal analysis, several have previously been associated with FEV_1_ and COPD. *MMP9* encodes the enzyme Matrix metalloproteinase-9 (MMP-9) which is implicated in the development of emphysema, mediating inflammation through extracellular matrix degradation and neutrophil recruitment [16]. *IL1RL1* encodes Interleukin 1 receptor-like 1 (IL1RL1), also known as suppression of tumorigenicity 2 (ST2), is a receptor for interleukin 33 (IL-33) and is part of the interleukin 1 receptor family. IL-33 and its receptor ST2 have a role in mediating immune responses and alveolar damage [17], and this pathway is a target for novel COPD therapies including itepekimab and astegolimab [18, 19]. *ALOX5AP* gene encodes arachidonate 5-lipoxygenase activating protein (ALOX5AP) that is essential in producing leukotriene B4 (LTB4), a molecule that drives inflammation by recruiting neutrophils to the lungs in COPD [20]. Elevated LTB4 levels in sputum are associated with worsened lung function and disease progression in COPD [21].

Only two genes (*NOV* and *AC009404.2*) overlapped across all three analyses, highlighting their potential significance in COPD and lung function regulation. *NOV*, encoding the CCN3 protein, is involved in inflammation and apoptosis. It has been identified as a biomarker for acute lung injury [22], suggesting its potential role in lung repair and disease pathogenesis. *AC009404.2* is annotated as a long non-coding RNA (lncRNA) located on chromosome 2, but it has not been studied in the respiratory literature.

The gene signature we generated for FEV_1_ change includes several genes previously associated with lung function, COPD, and related traits. *AKAP6*, which encodes A-kinase anchoring protein 6 (AKAP6), has been implicated in both COPD and lung function (FEV_1_/FVC ratio) through genome-wide association studies [23, 24]. Similarly, *GPR15*, which encodes G protein-coupled receptor 15, has emerged as a key gene associated with smoking status and more severe COPD [25, 26]. It acts as a chemoattractant receptor regulating immunity and T-cell migration, and its increased expression in smokers highlights its role in inflammation and smoking-related lung damage [27]. Single nucleotide polymorphisms in *SLC4A10* and *LAMA2*, which encode solute carrier family 4 member 10 (SLC4A10) and laminin subunit alpha 2 (LAMA2) respectively, have also been associated with smoking status and lung function (forced vital capacity) [28–30], further supporting their relevance in COPD pathophysiology. Other genes in the signature, such as Chromosome 12 Open Reading Frame 42 (*C12orf42)* and phosphatidylinositol glycan anchor biosynthesis, class L *(PIGL)*, have been associated with pulmonary arterial hypertension [31], smoking initiation [29], and FVC [24].

In addition to the genes previously associated with lung function and COPD, our gene signature was also associated with important COPD clinical traits related to FEV_1_ decline, such as severe exacerbations, exacerbation frequency, percent emphysema, and airway wall thickness. These associations suggest that the molecular factors influencing FEV_1_ decline may also be relevant to structural changes in the lungs and the severity of disease manifestations. Smoking cessation was associated with a significant reduction in the negative gene signature, highlighting its potential utility as a marker of inflammation or smoking-induced gene activity (see Supplementary material).

Interestingly, we were able to replicate most of the gene signature associations in another longitudinal COPD study, demonstrating consistent associations with FEV_1_ on a cross-sectional level, as well as with traits like airway wall thickness and exacerbation frequency. However, we were unable to replicate the association with FEV_1_ change. A possible reason is the differences in the time scale for lung function testing. In COPDGene, spirometry was performed every five years, whereas, in ECLIPSE, the measurements were taken annually, with a maximum follow-up of three years, which may have reduced the power of the analysis. This shorter follow-up may not have allowed enough time for a significant change in FEV_1_ to be detected.

Our study has several limitations. RNA-seq data were taken from blood samples, which may not perfectly capture the lung-specific aspects of COPD, However, they are well-suited for clinical applications and epidemiological research. This approach is particularly useful given that COPD has significant systemic comorbidities, including muscle loss, cardiovascular disease, and osteoporosis [32], highlighting the need for broader biomarker analysis beyond the lungs. The gene signature was generated using RNA-seq data from COPDGene, but the validation study used microarray data. Despite the differing technologies, the signature was replicated for many of the traits, which may imply better transferability of a gene signature biomarker as opposed to the expression of single genes. The sample size of Phase 3 RNA-sequencing was relatively small, which may have reduced our statistical power. Additionally, the longitudinal analysis was performed on only two-time points five years apart, which does not provide a full trajectory of FEV_1_ decline. Future studies will include larger sample sizes and more time points to improve the prediction of gene expression associations with lung function decline.

In conclusion, we identified genes associated with FEV_1_ decline in COPD, providing both previously known and novel insights into the molecular contributors to lung function decline. Our findings suggest that the identified genes may serve as potential biomarkers for COPD progression and targets for therapeutic intervention. Further studies are needed to validate these findings in larger cohorts, with more frequent longitudinal assessments, and in diverse populations, to better understand the clinical utility of these gene signatures in predicting disease trajectory for COPD patients.

## Supplementary Information

Supplementary Material 1

Supplementary Material 2

## Data Availability

The phenotype and RNA-seq data used in this study are available through the COPDGene Study under controlled access via dbGaP (accession numbers: phs000179.v6.p2, phs000765.v3.p2).

## Abbreviations

COPD: Chronic Obstructive Pulmonary Disease
FEV_1_: Forced Expiratory Volume in 1 s
FVC: Forced Vital Capacity
CPM: Counts Per Million
GSVA: Gene Set Variation Analysis
CS: Cross-Sectional
Pi10: Airway Wall Thickness
BD: Before Bronchodilator
WBC: White Blood Cells
