# Supplementary material for: Molecular determinants of lung function decline: a multi-level analysis of gene expression
Source: Respir Res. 2025 Dec 17;27:20. doi: 10.1186/s12931-025-03450-z (PMC12822344; doi:10.1186/s12931-025-03450-z)
Supplement: Supplementary file 1 — Supplementary Material 1 [file 12931_2025_3450_MOESM1_ESM.docx]

**Molecular Determinants of Lung Function Decline: A Multi-Level Analysis of Gene Expression**

Zaid W. Elhusseini, Omar Rafique, Min Hyung Ryu, Peter Castaldi, Don D. Sin, Ingo Ruczinski, Craig P. Hersh

**Supplemental methods**

**COPDGene Study**

COPDGene[1] (clinicaltrial.gov identifier: NCT00608764, registered on January 28, 2008) is a longitudinal observational study that enrolled participants aged 40–80 years with a smoking history of at least 10 pack-years. The cohort includes individuals of non-Hispanic White or African-American ancestry, both with and without chronic obstructive pulmonary disease (COPD), and excludes other known lung diseases aside from asthma. Demographic data, smoking status, medical history, post-bronchodilator spirometry, and chest CT scans were collected at each visit. The Phase 2 and 3 visits added complete blood counts and RNA-sequencing (RNA-Seq) samples. 3,819 participants in Phase 2 had both transcriptomic and phenotypic data. Of these, 3,802 had clinical data in Phase 1 and Phase 2, 2,043 had clinical data in Phase 2 and Phase 3, and 2,035 had clinical data in Phase 1 and Phase 3. A total of 437 participants had transcriptomic and clinical data in both Phase 2 and Phase 3. Ultimately, 435 participants were retained for analysis after excluding 2 individuals who each formed a unique batch, as ComBat-seq [2] requires at least two samples per batch for effective batch correction. To assess potential selection bias, we compared the demographic and clinical characteristics of the 435 subjects included in the longitudinal analysis to the remaining 3,384 Phase 2 participants who were excluded from Phase 3. No systematic differences were observed (Table S1). Details of the COPDgene study were previously published[1,3]. All participants provided written informed consent, and the study was approved by the relevant institutional review boards.

**Gene Expression Filtering and Normalization**

We performed four main analyses in R (version 4.3.2). First, we restricted our analyses to subjects with both gene expression (Phase 2 or Phase 3) and phenotypic data, and we combined these datasets. For each dataset, we calculated counts per million (CPM) values for every gene, and we retained only those genes for which at least 80% of the samples had a CPM greater than or equal to 1 to ensure that lowly expressed genes were excluded. Next, we used the edgeR:TMM[4] method to normalize the retained genes, accounting for library size differences among samples. Finally, we repeated this filtering and normalization procedure in each relevant dataset (e.g., Phase 2, Phase 3, and the combined set) to ensure consistency across analyses and facilitate direct comparisons between phases.

**Gene Expression Analysis**

We constructed a design matrix for each analysis, which included the relevant FEV_1_ measure (cross-sectional or change in FEV_1_), demographic factors (age, race, gender, smoking status), and cell-type proportions. Next, we fit linear models using the edgeR:voomLmFit function (a combination of the voom and lmFit functions in edgeR) [5] to estimate mean-variance trends followed by eBayes to identify significantly associated genes. To annotate Ensembl gene IDs, we used EnsDb.Hsapiens.v79 and get matching gene names with Ensembl gene IDs. Finally, we reported the top differentially expressed genes (p < 0.05).

**Gene signature:**

To generate the gene signature, we identified genes associated (pvalue<0.05) with FEV_1_ change across three intervals (Phase 1–Phase 2, Phase 1–Phase 3, and Phase 2–Phase 3) and classified them into two sets based on the direction of log fold-change (logFC), resulting in 17 positive (GS_Pos) and 3 negative (GS_Neg) gene signatures. We then performed Gene Set Variation Analysis (GSVA)[6] to assign each subject a score based on these gene sets. Finally, we assessed associations with various traits using linear regression for continuous traits or logistic regression for categorical traits, adjusting for age, race, gender, smoking status, and cell-type proportions, and compiled the significant findings into a single results table.

**ECLIPSE Study Replication**

The Evaluation of COPD Longitudinally to Identify Predictive Surrogate Endpoints (ECLIPSE) (ClinicalTrials.gov identifier: NCT00292552) was a multicenter, three-year prospective study that enrolled 2,747 participants [7]. Whole-blood RNA microarray data and tested traits were available for 627 of these individuals (Table S2). Participants were aged 40–75 years, had at least a 10 pack-year smoking history, no other known respiratory disease, and moderate to severe COPD (Global Initiative for Chronic Obstructive Lung Disease [GOLD] stage 2 or higher). The latter was defined as a post-bronchodilator forced expiratory volume in one second (FEV_1_) below 80% of predicted and an FEV_1_/forced vital capacity (FVC) ratio less than 0.7. Details of the ECLIPSE study were previously published [3,7]. All participants provided written informed consent, and the study was approved by the relevant institutional review boards.

To replicate our findings in ECLIPSE, we used the GSVA package [6]to compute each subject a score based on the previously selected gene sets. We then applied either linear regression or logistic regression, depending on the trait type. ECLIPSE has 2 sub-cohorts and 9 batches across samples. Each model controlled for relevant covariates (cohort, microarray batch, age, race, sex, smoking status, and cell-type proportions).

**Smoking status**

Smoking status was determined by a standardized questionnaire. Both COPDGene and ECLIPSE cohorts obtained the current smoking status by asking the subject whether they were smoking as of 1 month before the study visit when blood samples and clinical data were collected.

**Supplemental Analysis**

**Cross-sectional Analysis**

We performed a series of linear regression analyses, including cross-sectional analyses, FEV_1_ change analysis, and longitudinal analysis. In the cross-sectional analyses, the associations between gene expression and FEV_1_ were analyzed separately at Phases 2 and 3. We identified 740 genes in Phase 2 (Supplemental Table 2) and 225 genes in Phase 3 (Supplemental Table 3) that were associated with FEV_1_ at p < 0.05. Notably, only 11 genes overlapped between the two phases. This limited overlap may reflect variations in gene expression associated with COPD progression or other underlying biological changes occurring between phases. These findings suggest that cross-sectional analyses at different time points might capture distinct aspects of the disease process.

**Gene Signature Response to Smoking Cessation**

To assess whether smoking cessation influenced gene signature expression, we compared subjects who were current smokers at Phase 1 (P1) and remained smokers at Phase 2 (P2) (“CC”, n = 1,337) with those who were current smokers at P1 but had quit by P2 (“CF”, n = 468). Gene expression was measured at P2, capturing changes following smoking cessation. The Negative Gene Signature was significantly reduced in the CF group (β = –0.24, *p* < 2×10⁻¹⁶). The Positive Gene Signature was slightly elevated in CF compared to CC, though not statistically significant (*p* = 0.46). These findings align with biological expectations: the negative signature may reflect inflammation or smoking-induced damage, and its reduction suggests a response to smoking cessation, while the positive signature may represent recovery pathways.

**Predictive Performance of Gene Signatures for Rapid FEV_1_ Decline**

To evaluate whether gene signatures improve prediction of rapid FEV_1_ decline, we constructed receiver operating characteristic (ROC) curves comparing logistic regression models that added either the positive or negative gene signatures to a model including clinical covariates alone (Figure S1). The area under the ROC curve (AUC) for the covariates only model was 0.64. The positive or negative gene signature did not significantly increase the AUC. These findings indicate that the gene signatures do not improve the model’s ability to discriminate rapid decliners beyond standard covariates.

**Table S1.** Baseline characteristics of included and excluded Phase 2 participants.

| Clinical Characteristics | Not included in RNA-seq | Included in RNA-seq |
| --- | --- | --- |
| # Subjects | 3384 | 435 |
| Age (mean (SD)) | 65.51 (8.76) | 64.52 (8.17) |
| Sex = Male (%) | 1741 (51.4) | 214 (49.2) |
| Race = non-Hispanic White (%) | 2442 (72.2) | 323 (74.3) |
| FEV_1_ post-bronchodilator (mean (SD)) | 2.14 (0.84) | 2.31 (0.76) |
| Smoking status=Current(%) | 1284 (37.9) | 145 (33.3) |

Values are presented as mean (SD) or % for the indicated category. No systematic differences were found between groups.

**Table S2.** Demographic and Clinical Characteristics of ECLIPSE Subjects

| **Variable** | **Value (SD)** |
| --- | --- |
| Number of subjects | 627 |
| Age (SD) | 63.53 (6.32) |
| Male sex (%) | 66.2 |
| Current smokers (%) | 24.9 |
| FEV_1_ at Baseline (L) | 1.54 (0.79) |
| FEV_1_ at 1 Year (L) | 1.53 (0.79) |
| FEV_1_ at 2 Years (L) | 1.50 (0.77) |
| FEV_1_ at 3 Years (L) | 1.47 (0.78) |
| FEV_1_% predicted (%) | 50.25 (22.73) |
| FEV_1_Change (mL/year) | -26.69 (77.99) |


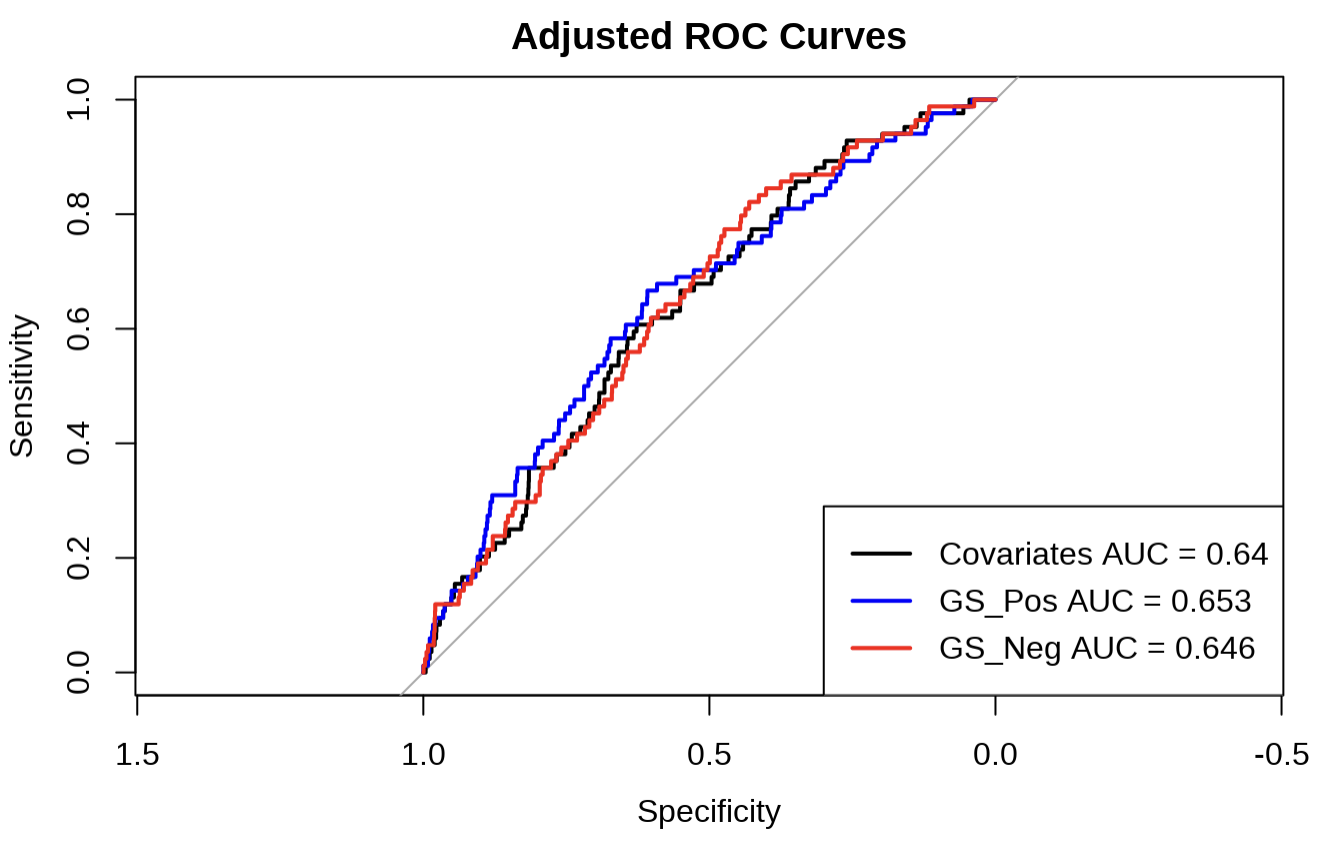


**Figure S6.** Receiver operating characteristic (ROC) curves.

Models including positive or negative gene signature did not significantly improve the area under the ROC curve (AUC) compared to a model including clinical covariates alone: DeLong’s test p=0.42 and p=0.66, respectively.

**References**

1 Regan EA, Hokanson JE, Murphy JR, *et al.* Genetic epidemiology of COPD (COPDGene) study design. *COPD*. 2010;7:32–43. doi: 10.3109/15412550903499522

2 Zhang Y, Parmigiani G, Johnson WE. ComBat-seq: batch effect adjustment for RNA-seq count data. *NAR Genom Bioinform*. 2020;2. doi: 10.1093/NARGAB/LQAA078

3 Ryu MH, Yun JH, Morrow JD, *et al.* Blood Gene Expression and Immune Cell Subtypes Associated with Chronic Obstructive Pulmonary Disease Exacerbations. *Am J Respir Crit Care Med*. 2023;208:247–55. doi: 10.1164/RCCM.202301-0085OC/SUPPL_FILE/DISCLOSURES.PDF

4 Robinson MD, Oshlack A. A scaling normalization method for differential expression analysis of RNA-seq data. *Genome Biol*. 2010;11:1–9. doi: 10.1186/GB-2010-11-3-R25/FIGURES/3

5 Robinson MD, McCarthy DJ, Smyth GK. edgeR: a Bioconductor package for differential expression analysis of digital gene expression data. *Bioinformatics*. 2010;26:139–40. doi: 10.1093/BIOINFORMATICS/BTP616

6 Hänzelmann S, Castelo R, Guinney J. GSVA: Gene set variation analysis for microarray and RNA-Seq data. *BMC Bioinformatics*. 2013;14:1–15. doi: 10.1186/1471-2105-14-7/FIGURES/7

7 Vestbo J, Anderson W, Coxson HO, *et al.* Evaluation of COPD Longitudinally to Identify Predictive Surrogate End-points (ECLIPSE). *Eur Respir J*. 2008;31:869–73. doi: 10.1183/09031936.00111707
